# The Aging, Community and Health Research Unit—Community Partnership Program for older adults with type 2 diabetes and multiple chronic conditions: a feasibility study

**DOI:** 10.1186/s40814-016-0063-1

**Published:** 2016-05-09

**Authors:** Maureen Markle-Reid, Jenny Ploeg, Kathryn Fisher, Holly Reimer, Sharon Kaasalainen, Amiram Gafni, Andrea Gruneir, Ross Kirkconnell, Sam Marzouk, Noori Akhtar-Danesh, Lehana Thabane, Carlos Rojas-Fernandez, Ross Upshur

**Affiliations:** 1 School of Nursing, Health Sciences Centre, McMaster University, 1280 Main Street West, Room 3N25B, Hamilton, ON L8S 4 K1 Canada; 2 School of Nursing, Health Sciences Centre, McMaster University, 1280 Main Street West, Room 3N25C, Hamilton, ON L8S 4 K1 Canada; 3 School of Nursing, Health Sciences Centre, McMaster University, 1280 Main Street West, Room 2J34A, Hamilton, ON L8S 4 K1 Canada; 4 Department of Clinical Epidemiology and Biostatistics, Centre for Health Economics and Policy Analysis, McMaster University, 1280 Main Street West, Room CRL-208, Hamilton, ON L8S 4 K1 Canada; 5 University Department of Family Medicine, University of Alberta, 6-40 University Terrace, Edmonton, AB T6G 2T4 Canada; 6 Guelph Family Health Team, Dawson Road Family Medical Centre, 83 Dawson Rd, Guelph, ON N1H 1B1 Canada; 7 Diabetes Care Guelph, Dawson Road Family Medical Centre, 83 Dawson Rd, Guelph, ON N1H 1B1 Canada; 8 School of Nursing, Health Sciences Centre, McMaster University, 1280 Main Street West, Room 3N28B, Hamilton, ON L8S 4 K1 Canada; 9 Department of Clinical Epidemiology and Biostatistics, St. Joseph’s Healthcare Hamilton, 3rd Floor, Martha Wing, Room H-325, 50 Charlton Avenue East, Hamilton, ON L8N 4A6 Canada; 10 School of Pharmacy, University of Waterloo, 200 University Avenue West, Waterloo, ON N2L 3G1 Canada; 11 Division of Clinical Public Health, Dalla Lana School of Public Health, 155 College Street, 6th floor, Toronto, ON M5T 3M7 Canada

**Keywords:** Nurse-led intervention, Older adults, Feasibility study, Diabetes self-management, Community-based care, Interdisciplinary

## Abstract

**Background:**

Few studies have examined the effectiveness of community-based self-management interventions in older adults with type 2 diabetes mellitus (T2DM) and multiple chronic conditions (MCC). The objectives of this study were to examine the feasibility of implementation in practice (primary) and the feasibility of study methods and potential effectiveness (secondary) of the Aging, Community and Health—Community Partnership Program, a new 6-month interprofessional, nurse-led program to promote diabetes self-management in older adults (>65 years) with T2DM and MCC.

**Methods:**

This study used a prospective one-group pre-test/post-test design. Participants were recruited from a specialized diabetes clinic. They received a median of three in-home/clinic visits by certified diabetes educators (CDEs) and attended a median of three group wellness sessions provided by the CDEs in partnership with a community-based seniors’ association. The primary outcome was the feasibility of the program (acceptability, fidelity, implementation barriers/facilitators). Secondary outcomes included the feasibility of the study methods (recruitment/retention rates and procedures, eligibility criteria, data collection and analysis methods) and potential effectiveness of the program based on 6-month changes in self-reported outcomes including self-management behavior (diet, exercise, self-monitoring), health status (quality of life, mental health), and costs of service use. Analysis of feasibility outcomes was primarily based on descriptive statistics. The potential effectiveness of the program was explored using different tests, with the results expressed using descriptive statistics and effect estimates (95 % confidence intervals).

**Results:**

In total, 45 (88 %) of 51 eligible persons consented to participate. Of these, 37 (82 %) completed the 6-month follow-up. Participants and providers viewed the program as acceptable and feasible. Participants had a higher SF-12 physical component summary score at 6 months compared with baseline (mean score difference 3.0, 95 % CI 0.2–5.8). Median costs for diabetes care increased over 6 months (reflecting inclusion of program costs), while other service costs either decreased or remained unchanged.

**Conclusions:**

This study offers preliminary evidence that the program was feasible to deliver and acceptable to participants and providers. Initial results suggest that the program may improve physical functioning. A randomized controlled trial is feasible, with some adaptations to the program and study methods that were identified from this feasibility study.

**Trial registration:**

Clinicaltrials.gov identifier: NCT01880476

**Electronic supplementary material:**

The online version of this article (doi:10.1186/s40814-016-0063-1) contains supplementary material, which is available to authorized users.

## Background

Diabetes is a rising global health concern. An estimated 150 million people worldwide were diagnosed with diabetes in the year 2000, which rose to 371 million in 2012 and is projected to reach 552 million by 2030 [1]. Of the three types of diabetes (gestational, type 1, and type 2), type 2 diabetes mellitus (T2DM) comprises 90–95 % of diabetes cases worldwide [2]. T2DM has a higher prevalence in older adults [3] and is caused by multiple interacting risk factors, many of which are modifiable [4]. Development of diabetes in at-risk older adults might be reduced by as much as 71 % through lifestyle changes, generating a net savings to the healthcare system [4, 5]. Accordingly, emphasis has been placed on the development of self-management interventions. A number of systematic reviews have found that self-management interventions are associated with improved clinical, behavioral, and psychosocial outcomes and health-related quality of life [6–16]. Evidence suggests that effective interventions incorporate behavioral and psychosocial strategies (e.g., motivational interviewing) [10], provide ongoing support [6, 15, 17], and use behavioral goal-setting to support self-management [18].

The studies in these reviews have typically omitted older adults with multiple chronic conditions (MCC) [19]. The omission of this group is important; about 60 % of older adults with T2DM have at least one comorbid condition and 40 % have 3 or more [4]. Thus, it is uncertain if the interventions are effective for these people, who are known to have poorer self-management and health-related quality of life (HRQoL), higher risk of diabetes-related complications, higher use of health services, and higher risk of institutionalization than those with T2DM alone [20]. In addition, they are at increased risk for geriatric conditions such as cognitive impairment [21], injurious falls [22], muscle weakness, urinary incontinence, frailty, and chronic pain [23], which complicate diabetes management and reduce HRQoL [24, 25].

Previous studies pertain primarily to interventions focused on treating biological factors rather than the full range of health conditions and contextual factors affecting diabetes self-management (e.g., psychological, social, and environmental factors) [15]. Similarly, the evidence-based guidelines derived from these studies adopt a single disease focus and fail to consider the management of T2DM within the context of MCC and fail to use patient-relevant outcomes (e.g., improving function or symptom burden instead of lowering HbA1C levels) [26–28]. Although prior studies have explored a variety of outcomes, most recommend further research regarding which outcomes can best assess the effectiveness of self-management interventions.

Emerging evidence suggests that group-based programs implemented in community settings and involving partnerships with diabetes care providers are feasible and effective and may better address the complex interplay of contextual factors [6, 29–31]. These programs enhance the current medicalized approach through the development of collaborations and partnerships that can provide a more holistic model of health and healthcare, ensure that programs are tailored to the unique needs of communities, and address the barriers to effective diabetes self-management [12, 32–35]. Recent US studies suggest that these programs may reach more people and be more self-sustaining, result in more efficient use of resources, and be acceptable to older adults with MCC [36–43]. Yet most of these studies have not involved patients, caregivers, or clinicians in the design of the interventions and identification of relevant outcomes, which is likely to limit their ability to address client needs and adaptability to local settings. More information is also needed on adapting community-based interventions to individual settings, the effectiveness of programs in key patient subgroups such as older adults with MCC, and program costs [29].

A community-based program of diabetes self-management for older adults with T2DM and MCC that results in efficient use of resources remains elusive [44]. New and innovative models of care need to be developed and tested to improve the physical, mental and social functioning of this group [32, 45, 46]. We also need to determine the best outcomes to assess the effectiveness of these models. Continuing research on MCC suggests that interventions for older adults with MCC need to be comprehensive, participatory, client-centered, multifaceted, and involve diverse partnerships [20]. This feasibility study explored a program encompassing these key elements that was designed for community-living older adults with T2DM and MCC. It aimed to determine the feasibility of a future multisite randomized controlled trial (RCT) to evaluate the effectiveness and costs of the program. The presentation of the results from this study follows the recommendations from two guidance documents: the Additional file 1 guidelines for reporting observational studies [47] and the recommendations for how to report the results of pilot and feasibility investigations [48].

### Objectives

The overarching goal of this feasibility study was to investigate the feasibility of a larger RCT that will examine the effectiveness of the Aging, Community and Health (ACHRU)—Community Partnership Program, an interprofessional, nurse-led program for community-living older adults with T2DM and MCC. Primary and secondary study objectives were designed to achieve this goal. The primary objective was to determine the feasibility of implementation of this program in practice. The secondary objectives were to (1) determine the feasibility of the study methods used to evaluate this program, (2) obtain emergent evidence of the potential effectiveness of this program based on changes over 6 months in self-management behavior, health status, and costs of use of health and social services, and (3) determine the most appropriate primary outcome measure for the larger RCT.

## Methods

### Study design

Because of the complexity of evaluating health service interventions, a mixed-method design (quantitative and qualitative) was used to examine the interaction between the community care context, program implementation, and outcomes [49]. The feasibility study used a single arm, pre-test/post-test design. This design seemed to be well suited to our study, which was investigating a new and innovative program for which little information existed on the feasibility of the program and the ability to carry out a large-scale trial. Our interests in this early investigative phase were focused on maximizing program feedback, assessing the feasibility of program implementation and study methods, and exploring the performance of the outcome measures, rather than on developing definitive estimates of program effectiveness. Six months was selected for the program length for the feasibility study and larger RCT, which is supported by previous research [50]. The research team preferred a longer time to obtain more definitive information on program feasibility and sustainability, but budget constraints and provider preferences dictated the choice of 6 months. Accordingly, our secondary objective (#2) focused on looking for *preliminary* evidence of *potential* program effectiveness.

Common threats in pre-test/post-test designs of intervention studies include the placebo effect, history, statistical regression to the mean, and confounding with usual care. Methods were employed to minimize as many of these threats as possible. To minimize the placebo effect, participants were permitted to choose the number of in-home visits and group sessions, which reinforced the perception that individual needs were different and more was not necessarily better. Also, informed consent stressed that usual care would remain in place, because it was not clear that there were effects beyond those associated with usual care (hence the need for the study). Threats posed by history were minimized by using an intervention period of 6 months (longer time periods increase the likelihood of other changes occurring) and ensuring that post-intervention assessments were completed within 2 weeks of the end of the intervention. Statistical regression to the mean effects were reduced because participants were chosen on the basis of inclusive criteria considered representative of the broader target population (older adults with diabetes and multiple chronic conditions), rather than chosen on the basis of extreme instrument scores (thus representing a restrictive sub-group of the target population).

### Participants and setting

This was a collaborative project between the Aging, Community and Health Research Unit (ACHRU) at McMaster University (Hamilton, Ontario, Canada) and managers and practitioners in a seniors’ association and a specialized diabetes clinic located in southcentral Ontario, Canada. The goal of ACHRU is to promote optimal aging at home for older adults with MCC and to support their family caregivers. ACHRU is mandated by its funders to design, evaluate, and translate new and innovative interprofessional community-based programs to improve access to healthcare, health-related quality of life, and health outcomes in this population, while reducing costs. The study location was a city of moderate size (population 120,000–150,000), where older adults represented 13 % of the total population. The Diabetes Education Centre (DEC) was part of a larger primary healthcare practice. The DEC recruited study participants and provided the trained diabetes educators who partnered with representatives from a community-based seniors association to deliver the program.

Study participants were 65 years or older, enrolled in the DEC or receiving diabetes-related services from a family health team (i.e., a team of family physicians, nurse practitioners, registered nurses, social workers, dietitians, and others who work together to provide primary healthcare in the community) within the previous 24 months, diagnosed with ≥2 chronic conditions in addition to T2DM, competent in English (or with an interpreter available), and not planning to move away from the community within 6 months of study enrollment.

### Screening for eligibility and enrolment

Recruitment took place between July and December, 2013. Trained DEC staff identified potential participants based on the inclusion criteria and then telephoned them to obtain verbal consent to be contacted by a research assistant from ACHRU. A research assistant conducted an in-home interview to obtain written informed consent and complete the baseline questionnaires. Older adults were deemed to be mentally competent and thus eligible for the study if they scored >20 on the Montreal Cognitive Assessment (MoCA) [51]. All eligible and consenting participants were assigned to the program.

### Program

This 6-month program was complex and consisted of several interacting components including (1) home visits by a registered nurse (RN) and registered dietitian (RD) from the DEC, (2) monthly group sessions for participants, hosted by the seniors’ association in partnership with the DEC, (3) monthly nurse-led case conferences for team members, and (4) nurse-led care coordination. The combination of a RN and RD aligned well with usual care, as both were already delivering diabetes services at the DEC. Community nurses are in an ideal position to lead an interprofessional team given their scope of practice. A number of RCTs also show the effectiveness of interprofessional, nurse-led interventions involving community-dwelling older adults with MCC [50]. Nurse-led care coordination typically involves a trained nurse care manager who works collaboratively with primary care, home care, and other members of the interprofessional care team.

Participants could decline any number of home visits or group sessions, and all participants continued to have access to the programs and services normally offered by the DEC and the seniors’ association (usual care). The seniors’ association and its volunteer network was an excellent foundation for the program, given their exceptional reach into the community and history of implementing a wide variety of successful programs for seniors, including the long-established diabetes support group. The DEC was an equally capable partner, with certified diabetes educators (CDEs) already delivering a diabetes self-management education (DSME) program aimed at supporting informed decision-making, self-care, and problem-solving to improve clinical outcomes, health status, and quality of life.

The program protocol was developed using the guidelines for developing complex interventions [52]. It was based on best practice guidelines for diabetes [4]; empirical evidence related to diabetes care for older adults with T2DM; and qualitative interviews with older adults, family caregivers, and community service providers. It was also grounded in Bandura’s Social Cognitive Theory [53], which recognizes the central role of self-efficacy in behavior. The program components were designed to capture key constructs in Bandura’s model. For example, key sources of self-efficacy (e.g., social modelling, mastery) were the target of group sessions and home visits. The program addressed the full range of diabetes self-care activities within the context of MCC but was inherently flexible so that it could be shaped by participants and tailored to their needs.

The program also consisted of several components not normally included in usual care. One such component was the coordination of community-based services. The program was provided by CDEs from a specialized diabetes clinic in partnership with a program coordinator and physical activity leader from a seniors’ association. Three peer support volunteers recruited by the seniors’ association assisted with delivery of the group session component of the program. Another key component was holistic, client-centered care, facilitated by home visits to more fully understand the participant’s socio-personal context. Each participant was offered four in-home visits by the RN and RD over 6 months; the number of visits was based on previous research [54] and feasibility in a larger RCT if the results of this feasibility study were supportive. To enhance social support and provide further opportunities for education and learning, the participants were invited to take part in six monthly group sessions held at the community centre. The 3-h group sessions included a light meal, socialization time, gentle exercise, and an interactive and flexible diabetes education and discussion component. All participants were required to have an assessment by a registered kinesiologist to identify potential exercise restrictions before attending their first group session.

The program emphasized team communication and collaboration. Monthly, the RN, RD, program coordinator, and physical activity leader met for a case conference to develop a client-centered and evidence-based plan of care for each participant. Case conferences provided an opportunity to share observations about participants’ strengths, challenges, and goals related to diabetes self-management; identify needs for other health professionals or community services; and prepare for upcoming group sessions. The RN provided care coordination; facilitated access to services and supports across the care continuum; and coordinated communication among the participant, caregiver, program team, and primary care providers.

The program was implemented using a multifaceted approach. First, ACHRU investigators held an 8-h workshop with the RN, RD, physical activity leader and program coordinator together. Then, separate 8-h workshops were held for two groups: (1) the RN and RD and (2) the physical activity leader and program coordinator. The three peer support volunteers attended a 4-h workshop. To support program fidelity, a manual was prepared describing the program, and all team members were trained in the methods and content of the program. To monitor implementation and discuss challenges, the principal investigator and/or research coordinator met with the program team at monthly “outreach” meetings.

### Outcome measures

Table 1 provides a summary of the outcomes, measures/approaches, and analyses corresponding to the primary and secondary study objectives.

**Table 1:** Summary of outcomes, measures/approaches, and methods of analysis

Objective	Outcomes	Measures/approaches	Methods of analysis
1. Feasibility of program implementation	Client acceptability	-Semi-structured interview (at 6 months)	-Content analysis
		-% of participants that did not die or transfer to long-term care and completed the 6-month program	-Descriptive statistics
		-% of completers that had at least 1 home visit	-Descriptive statistics
		-% of completers that had at least 1 group session	
	Provider acceptability	-Focus group interview (at 6 months)	-Content analysis
	Peer support volunteer acceptability	-Focus group interview (at 6 months)	-Content analysis
	Implementation barriers and facilitators	-Focus groups (providers)	-Content analysis
		-Semi-structured interviews (clients)	
	Fidelity	-Compare log (visits, case conferences, group attendance) to fidelity checklist	-Rating of compliance
			-Descriptive feedback
2. Feasibility of study methods	Eligibility	-No. of individuals screened and found eligible to participate in study	-Descriptive statistics
	Recruitment	-No. of eligible participants enrolled in study	-Descriptive statistics
	Retention	-% of enrolled participants that completed 6-month program	-Descriptive statistics
	Representative	-Comparison of completers and non-completers (characteristics: sociodemographic, self-management, health status, cost characteristics)	-Descriptive statistics
	Data collection and analysis	-Length of interview	-Content analysis
		-Clarity and acceptability of questions	
		-Applicability of questions to clients	
		-Ease of data collection	
		-Follow-up on missing or inconsistent response data	
		-Focus groups and semi-structured interviews for problems relating to data requested or issues affecting data analysis	
3. Change in client outcomes	Self-management behavior	-SDSCA^a^ total scale, sub-scale items	-Mean (SD), 95 % CI for mean score difference
	Health-related quality of life	-PCS^c^ score from SF-12^b^ (physical)	-Mean (SD), 95 % CI for mean score difference
		-MCS^d^ score from SF-12^b^ (mental)	
	Depression	-CES-D^e^ score	-Mean (SD), 95 % CI for mean score difference
			-Descriptive statistics (cut-off analysis)
	Anxiety	-GAD-7^f^ score	-Mean (SD), 95 % CI for mean score difference
			-Descriptive statistics (cut-off analysis)
	Glycated hemoglobin	-HbA1C measure (% sugar in blood)	-Mean (SD), 95 % CI for mean difference
	Hypoglycemic episodes (blood glucose <4 mmol/L)	-Number (proportion) of clients in each category: not sure, never or hardly ever, more than once a month, more than once a week, daily or almost daily	-Descriptive statistics
	Diabetes medication use	-Number (proportion) of clients in each category: increased medications, decreased medications, no change in medications	-Descriptive statistics
	Costs	-6-month costs by service type	-Median costs
4. Primary outcome for full RCT	SDSCA, PCS, HbA1C	-Applicability to clients	-Comprehensive performance evaluation of candidate outcome measures
		-Face validity	
		-Ease of collection/completion	

^a^SDSCA = Summary of Diabetes Self Care Activities Scale

^b^SF-12 = Short-Form Health Survey (12 questions)

^c^PCS = Physical Component Summary Score (from SF-12)

^d^MCS = Mental Component Summary Score (from SF-12)

^e^CES-D = Center for Epidemiologic Studies Depression Scale

^f^GAD-7 = Generalized Anxiety Disorder Scale (7 questions)

#### Feasibility of the program

Qualitative feedback was obtained at 6 months from providers, peer support volunteers, and participants regarding program acceptability and implementation barriers/facilitators. The 6-month focus group for providers was attended by the RN and RD from the DEC and the program coordinator and physical activity leader from the seniors’ association. The 6-month focus group for peer support volunteers was attended by the three volunteers. Six-month exit interviews were held with all participants that completed the program. All three groups were asked open-ended questions about the program regarding its perceived benefits, how it should be changed, what they liked and did not like, and implementation barriers and facilitators. Sessions were audio-taped and transcribed verbatim. Two quantitative measures were used to assess the program acceptability to participants: (1) percentage of participants that did not die or transfer to long-term care who completed the program; (2) percentage of completers who had at least one home visit and group session (“engagement rate”).

A recent systematic review found that program fidelity for diabetes self-management education (DSME) programs remains largely under-investigated [55]; therefore, we had little specific guidance in assessing program fidelity. Carroll et al’s [56] generic fidelity implementation model was used to develop a checklist that employed a simple, present/absent response format (Table 5, Appendix). Previous research suggests this format is easier to use and more reliable than complex frequency scales [57, 58]. One researcher reviewed source documents (e.g., visit and case conference records, training manuals) to assess the elements on the checklist.

#### Feasibility of the study methods

Eligibility was defined as the percentage of clients screened that were eligible to participate in the study. Our target was ≥50 %, based on the assumptions that 67 % of T2DM clients would have two or more other chronic conditions rate observed in recent study of Ontario older adults with diabetes, unpublished report by Griffith et al. 2015 and 75 % of these would be deemed eligible. Recruitment was defined as the percentage of eligible clients that enrolled in the study. We set a (somewhat arbitrary) target of ≥50 % for this outcome. Retention was defined as the percentage of enrolled clients that completed the 6-month program. We set a target of ≥80 % for the retention rate, based on the common view that bias is a concern if attrition exceeds 20 % [59]. Representativeness was defined as the absence of substantial differences between completers and non-completers on a range of characteristics collected at baseline.

Questionnaires, administered by trained interviewers, were used to collect data at baseline and 6 months. Inter-rater reliability was established prior to data collection. At baseline, we also collected sociodemographic data and medical history. The research assistants provided feedback on interview length, clarity and acceptability of interview questions, applicability of questions to participants, and ease of collecting data. Researchers reviewed the data collected, explored the reasons for missing or inconsistent responses, and reviewed the results from the focus groups and interviews for indications of important issues relating to data collection or analysis.

#### 6-month change in outcome measures

Self-management behavior was measured using the Summary of Diabetes Self-Care Activities (SDSCA) scale [60]. The SDSCA is well-validated, widely used, and recommended for evaluating DSME interventions [61, 62]. Responses capture the number of days in the prior week that self-care activities were performed (e.g., diet, exercise, monitoring of glucose levels and feet). Total scores range from 0 to 70 and higher scores indicate higher levels of self-management activity.

Health-related quality of life (HRQoL) was measured using the SF-12 [63]. The SF-12 is well-validated and able to distinguish between groups of clients with known differences in physical and mental health in a variety of populations. The physical component summary score (PCS-12) and mental component summary score (MCS-12) were used to summarize the data. Both scores range from 0 to 100 and higher scores indicate higher levels of HRQoL [64].

Depressive symptoms were measured using the original 20-item CES-D scale [65]. Total scores range from 0 to 30 and a higher score indicates a higher risk of depressive symptoms. A cut-off of 16 was used to identify clients with clinically relevant depressive symptoms [66]. Anxiety was measured using the GAD-7, which is based on the DSM IV criteria for generalized anxiety disorder [67]. GAD-7 has been used in research as a generic measure of anxiety symptoms and has been found to have good internal consistency and good convergent validity with other anxiety and disability scales [68–71]. Total scores range from 0 to 21 and higher scores indicate a higher level of anxiety A cut-off of 5 was used as the threshold for identifying the presence of anxiety disorder [69].

The two measures of metabolic control included in our study were glycated hemoglobin (HbA1C) and the frequency of hypoglycemic episodes. HbA1C serves as a marker of the average glucose concentration in the blood over a period of 2–3 months and can be expressed as a percentage or as a value in millimoles per mole (our results are expressed as a percentage) [72]. To measure blood glucose level, we asked the participants to report the frequency of hypoglycemic episodes over the previous 6 months. We defined hypoglycemic episodes as blood glucose levels <4 mmol.

We compared the use of all diabetes medications before and after the program, to explore the effects of changes in self-management behavior. The effects could be increased use of medications as a result of increased compliance or decreased use of medications as a result of improvements in other behavioral practices (e.g., diet, exercise, smoking). This was captured by comparing the proportion of participants before and after the program who increased, decreased, or did not change their use of diabetes medications.

The costs of use of health and social services were determined using the Health and Social Services Utilization Index (HSSUI) (unpublished paper, Browne et al. 2006). The HSSUI assumes a societal perspective, has been previously assessed for reliability and validity [73, 74], and is acknowledged as one of the few empirically validated measures of ambulatory utilization [75]. The HSSUI asks respondents about their use of a comprehensive range of health and social services. Inquiries were restricted to the reliable duration of recall (6 months for hospitalizations and physician visits, 2 days for prescription medication use). The total 6-month costs were derived from the product of “quantity” data reported from the HSSUI and 2014 “price” data obtained from a costing manual prepared by our research team [76]. Health service costs included the costs of delivering the program (e.g., home visits, group sessions, case conferences). Costs for each service type were expressed as a median cost (CAD). We also report median total health service use costs, including and excluding hospitalization costs.

#### Primary outcome for RCT

The candidate measures for the primary outcome in the larger RCT were the SDSCA, PCS from the SF-12, and HbA1C level. The criteria used to evaluate these measures included their applicability and relevance to the older adult study participants, face validity, and ease of data collection. Research assistants provided feedback on these criteria. Researchers also reviewed the data collected for each outcome measure, explored reasons for missing or inconsistent responses, and considered the responsiveness of each measure over the 6 months.

### Sample size

The primary objective of this feasibility study was to determine the feasibility of the program, thus we focused on determining the number of participants that we could recruit over the 6 months at the study site, to provide information on the number of sites and time needed to recruit the sample required for the RCT. We also thought the 6-month time frame would generate a sample of between 20 and 40 participants, the size that Hertzog [77] found sufficient for pilot and feasibility studies that varied in terms of purpose, desired precision, and effect sizes.

### Analytical methods

The last column of Table 1 indicates the analytical methods applied to the measures used to evaluate the primary and secondary study objectives. Content analysis was used to analyze qualitative data from focus groups and interviews [78], and the data were categorized according to acceptability of the intervention and facilitators and barriers to implementation. Participant characteristics and outcomes were summarized using descriptive statistics: mean (standard deviation) for continuous variables and number (percent) for categorical variables. Descriptive statistics were used to compare completers and non-completers. Analysis of feasibility outcomes was primarily based on descriptive statistics. The potential impact of the intervention on continuous clinical outcomes (scale/sub-scale scores for SDSCA, PCS, MCS, CES-D, GAD-7; HbA1C levels) was reported as estimates of effect (95 % confidence interval). Descriptive statistics described the results for categorical outcomes (CES-D and GAD-7 cut-off analyses). Differences in median costs for each service for the 6 months prior to baseline and the 6-month program period were calculated, and the change in each was compared to the hypothesized direction (increase in program costs, lower or no change in all other service costs). Descriptive statistics were used for comparing glycemic control measures. A complete case analysis was done. All statistical analyses used SAS Version 9.3 for Windows and were reported to one decimal place. All qualitative data analyses used manual rather than computer-assisted methods.

### Ethics

This study was conducted in accordance with the Tri-Council Policy Statement: Ethical Conduct for Research Involving Humans [79]. Ethics approval was obtained from the Hamilton Integrated Research Ethics Board (#13-377) and was renewed yearly as required. All participants provided written informed consent.

## Results

### Baseline demographic profile and diabetes-related clinical characteristics

Baseline characteristics of the participants are displayed in Table 2. There were slightly more females than males (56 % versus 44 %), 33 % were 65–69 years of age and 14 % were at least 80 years of age, and almost half (47 %) had some post-secondary education. Almost 70 % of the sample had an annual gross household income level below $40,000; 28 % reported an income <$20,000. About 58 % of the sample lived with a spouse or other person(s). Diabetes was a newly diagnosed condition (<1 year) for 25 % of participants, while 31 % were living with diabetes for >10 years; and 63 % took at least one diabetes medication. Comorbidity was common, with 92 % of the sample having 5 or more other conditions. Three comorbid conditions were highly prevalent: hypertension (86 %), dyslipidemia (78 %), and arthritis (75 %). Ninety-four percent were taking at least 3 prescription medications and 58 % were taking 6 or more. Hearing loss was also quite common, affecting almost half (47 %) of the study sample. Depression or anxiety was reported by 33 % of the sample.

**Table 2:** Baseline demographic profile and diabetes-related clinical characteristics (*n* = 36)

Item	Categories	*n* (%)
Gender	Male	16 (44.4)
	Female	20 (55.6)
Age (years)	65–69	12 (33.3)
	70–74	15 (41.7)
	75–79	4 (11.1)
	80+	5 (13.9)
Education	Less than high school	14 (38.9)
	High school	5 (13.9)
	Post-secondary	17 (47.2)
Income (gross, annual)	$10,000–$20,000	10 (27.8)
	$20,000–$40,000	15 (41.7)
	$40,000–$70,000	8 (22.2)
	$70,000+	3 (8.3)
Marital status	Married, common law	20 (55.6)
	Never married	3 (8.3)
	Divorced, separated	5 (13.9)
	Widowed	8 (22.2)
Living status	Lives alone	15 (41.7)
	Lives with spouse or others	21 (58.3)
Time since diabetes diagnosis	Less than 1 year	9 (25.0)
	1–5 years	10 (27.8)
	6–10 years	6 (16.7)
	More than 10 years	11 (30.6)
Number of diabetes medications (oral and insulin)	No medications	13 (36.1)
	1 medication	10 (27.8)
	2 medications	10 (27.8)
	3 medications	3 (8.3)
Number of total medications (diabetes and non-diabetes)	0–2	2 (5.6)
	3–5	13 (36.1)
	6+	21 (58.3)
Number of chronic conditions	2–4	3 (8.3)
	5–7	19 (52.8)
	8+	14 (38.9)
Common conditions (sample prevalence ≥25 %)	Hypertension	31 (86.1)
	Dyslipidemia	28 (77.8)
	Arthritis (osteoarthritis or rheumatoid arthritis)	27 (75.0)
	Hearing loss	17 (47.2)
	Depression or anxiety	12 (33.3)
	Cataracts	11 (30.6)
	Peripheral neuropathy/poor circulation	11 (30.6)
	Acid reflux/hiatal hernia	10 (27.8)
	History of heart attack	9 (25.0)

### Primary objective: feasibility of the program

#### Acceptability of program

Table 6 (Appendix) provides the feedback data from the focus group session and monthly research meetings with the providers, focus group session with peer support volunteers, and participant interviews. The feedback from providers was positive. The program enabled them to better understand the health status and challenges facing clients, as a result of the multiple opportunities for interprofessional collaboration, the synergistic effects of different program components, and the unique insights gained from seeing clients in their home environment. There was evidence of “buy in” for the program, with providers envisioning ways to implement the program in practice (e.g., using closed group sessions to strengthen relationships), anticipating the disappointment that clients would experience in transitioning from home visits back to clinic visits (usual care) at the end of the study, and suggesting that the group sessions be opened up to a close family member or friend to provide further support to participants. Providers and peer support volunteers both commented on the importance of the meal component of the group sessions, which enabled social interaction.

Feedback from participants mirrored these positive comments. Participants noted that the providers were competent, knowledgeable, and empathetic. They mentioned the motivational aspects of the group sessions and the value of home visits for both the providers and themselves. They, too, commented on the benefit of having a close family member or friend attend the group sessions. Further evidence of the acceptability of the program to participants is provided by data on the uptake of the program components (“engagement rate” = ≥1 episode, with an episode defined as a home visit or attendance at a group session). Three participants died or transferred to long-term care, leaving 42 participants in the study that had the choice to remain in or drop out. Thirty-seven participants completed the 6-month interviews, including one participant who refused all components of the program. Therefore, 36 of the 42 participants chose to receive the program and we viewed this as the best indicator of acceptability, which resulted in an acceptability rate of 86 % (36/42). The engagement rate for study completers (*n* = 37) was 97 % for home visits (36/37) and 76 % for group sessions (28/37). Engagement rates were slightly lower for females than for males, with rates of 95 % versus 100 % for home visits and 75 % versus 81 % for group sessions.

Certain aspects of the program were found to be challenging. The RN leading the program indicated that the administrative burden was high and suggested either more administrative support or more even distribution of duties across team members. Other challenges raised by the providers included the limited access to resource materials at home visits, ensuring that group sessions focus on group (not individual) issues and encourage participation by all members, and ensuring that the physical exercise component of group sessions is suitable for all participants and integrated with other program components. All three groups indicated that 3 h was sufficient or perhaps too long for the group sessions.

#### Facilitators and barriers to program implementation

The potential facilitators and barriers of program implementation were identified from the feedback received from the three groups. Important facilitators included adequate support for administrative activities, the option of home or clinic visits for participants, the motivational and facilitation characteristics of providers, flexibility and appropriate length for group sessions, maintaining a group (not individual) focus at group sessions, and attendance of a close family member or friend at the group sessions. Important barriers included group sessions in excess of 3 h, group sessions that were dominated by a small number of participants, physical exercises that could not be adapted to individual capabilities, and lack of administrative support. There was low uptake of transportation services offered by the program, suggesting that this was not a significant barrier to participation.

The feedback obtained from the focus group sessions and interviews was instrumental in identifying modifications to the program that would facilitate its successful implementation in a RCT. The last column of (Appendix) Table 6 maps feasibility study feedback to program changes proposed for the full RCT. Positive feedback did not require changes to the program, but we proposed protocol changes to address all remaining comments (see “Discussion” section below).

#### Program fidelity

Training protocols, manuals, and workshops were delivered to all providers, recruiters, and research assistants. The program was tailored so participants had the option of declining some or the entire program; however, only one participant declined all program components. Clients received a median of 3 home visits (11 % 1–2 visits, 86 % 3 visits, 3 % 5 visits) and attended a median of 3 group sessions (33 % 1–2 sessions, 45 % 3–4 sessions, 22 % 5–6 sessions). The engagement rate was 97 % for the home visits and 76 % for the group sessions. Regarding the *potential* effectiveness of the program (used in judging treatment enactment), significant changes in the outcome measures over 6 months were not expected because the feasibility study was underpowered and the program time frame was relatively short. The significant increase in HRQoL (PCS) observed in the feasibility study (see below) was unexpected and taken as a *preliminary* yet promising indication of the *potential* effectiveness of the program.

Although modifications to the program components were identified from the feedback received from providers, volunteers, and participants, the program was delivered as intended and was well received by all three groups. The variation in dose received by participants was appropriate for a tailored, client-driven program. The overall conclusion was that the program was delivered as planned.

### Secondary objective: feasibility of study methods

#### Participant flow

**Fig. 1:**
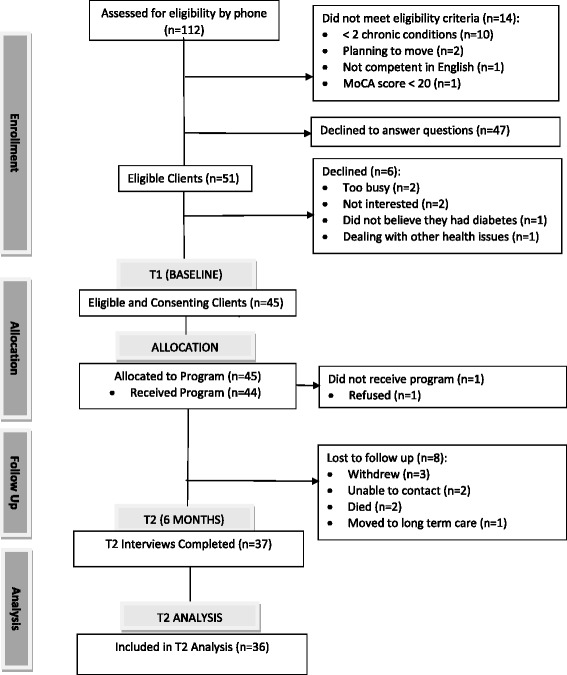
Study flow diagram

The measures used to evaluate the feasibility of the study methods include the eligibility, recruitment, and retention rates, derived from the CONSORT flow diagram (Fig. 1). Forty-seven of the 112 potentially eligible participants refused to answer the screening questions. Of the remaining 65 clients, 14 did not meet the eligibility criteria, resulting in an eligibility rate of 78 % (51/65) (target of 75 %). Six clients declined participation in the study, resulting in a recruitment rate of 88 % (45/51) (target of 50 %). Eight participants were lost to follow-up, resulting in a retention rate of 82 % (37/45) (target of 80 %). These results show that our targets were met for eligibility, recruitment, and retention rates.

One of the participants who completed the 6-month interview did not receive the program. Because 6-month outcome measures were available for this participant, she was included in the analysis. One participant was excluded from the analysis because he misinterpreted the self-management instrument.

#### Study sample representativeness

We compared completers and non-completers on baseline sociodemographic characteristics, diabetes history, MCC, number of diabetes and total medications, self-management behavior, and health status. Some differences were observed; specifically, completers were more likely to

- live alone (58 % versus 0 %);
- have higher household incomes (0 % below $10,000 versus 50 %);
- take fewer diabetes medications (8 % took 3 diabetes medications versus 87 %); and
- have fewer strokes or transient ischemic attacks (6 % versus 38 %).

#### Data collection and analysis procedures

Finally, we examined feedback from the research assistants and researchers on the data collection and analysis procedures. Table 7 (Appendix) provides the feedback from Research Assistants and researcher review of the response data and feedback from the focus group sessions. Research assistants indicated that the length of time for the baseline and 6-month interviews was 1.5–2 h and 1.5 h, respectively. They reported that the MoCA was time consuming, and the researcher team questioned the appropriateness of this instrument, given its limited role in the study (validating informed consent). Providers reported that falls were a concern for many participants and suggested that a falls assessment might be appropriate. Several concerns arose with administering the SDSCA, including its face validity with clients, the inapplicability of several questions (e.g., glucose monitoring, medications), and its potential for misinterpretation (which resulted in the removal of one client from the analysis). Regarding HbA1C measures, the main concerns were relevance (e.g., appropriateness relative to other measures, consistency with participant values/goals) and ensuring that the timing was more precise relative to the baseline and 6-month benchmarks. Researchers also felt that monthly receipt of the program documentation was too infrequent to facilitate tracking. The last column of (Appendix) Table 7 maps the issues raised to suggested changes to the data analysis and collection methods for the RCT (see “Discussion” section below).

### Secondary objective: changes in outcome measures at 6 months

#### Self-management behavior, HRQoL, depressive symptoms, anxiety, and HbA1C

Table 3 shows the values for the outcome measures at baseline and 6 months. At baseline, the mean score (standard deviation) for diabetes self-management was 33.8 (11.2) and HbA1C level was 7.2 % (1.1 %), with higher levels of self-management and HbA1C in females than in males. Mean (standard deviation) scores for depression and anxiety were 7.7 (7.6) and 2.7 (2.9), respectively, with females having higher scores for both measures. Nine percent of the sample were on or above the CES-D threshold for depressive symptoms (16+) and 28 % were on or above the GAD-7 threshold for anxiety disorder (5+). The mean PCS score of 41.3 (12.5) resulted from mean scores of 43.9 (12.1) for males and 39.0 (12.7) for females. The mean MCS score of 55.9 (6.9) resulted from mean scores of 55.7 for males (6.2) and 56.3 (7.7) for females.

**Table 3:** Baseline and 6-month scores for clinical outcomes

Scale/item	*N*	Baseline (T1) score mean (SD) or *n* (%) above/below cut-off	6-month (T2) score mean (SD) or *n* (%) above/below cut-off	Baseline to 6-month (T2–T1) score difference mean (SD)	95 % CI for mean score difference
General diet—1	36	5.8 (1.6)	5.5 (1.8)	−0.3 (1.9)	−0.9–0.4
General diet—2	36	5.4 (1.6)	5.5 (1.6)	0.1 (1.9)	−0.5–0.8
General diet subscale	36	5.6 (1.5)	5.5 (1.6)	−0.1 (1.8)	−0.7–0.5
Special diet—1	36	5.3 (2.6)	5.6 (1.2)	0.3 (1.5)	−0.3–0.8
Special diet—2	36	4.1 (2.2)	3.6 (2.4)	−0.5 (2.7)	−1.3–0.5
Special diet subscale	36	4.7 (2.0)	4.6 (1.6)	−0.1 (1.4)	−0.6–0.4
Exercise—1	36	4.2 (2.6)	4.2 (2.4)	0.0 (2.7)	−0.9–0.9
Exercise—2	36	2.3 (2.8)	1.8 (2.4)	−0.5 (2.5)	−1.4–0.4
Exercise subscale	36	3.2 (2.3)	3.0 (2.0)	−0.2 (2.3)	−1.0–0.5
Foot care—1	36	5.3 (2.4)	5.4 (2.4)	0.1 (2.1)	−0.7–0.7
Foot care—2	36	1.4 (2.7)	2.1 (3.00)	0.7 (2.9)	−0.4–1.6
Foot care subscale	36	3.4 (2.1)	3.7 (2.3)	0.3 (1.9)	−0.3–1.0
SDSCA total scale	36	33.8 (11.2)	33.6 (9.8)	−0.2 (8.9)	−3.2–2.9
PCS-12	35	41.3 (12.5)	44.3 (11.2)	3.0 (8.0)	0.3−5.8
MCS-12	35	55.9 (6.9)	55.4 (8.4)	−0.6 (8.4)	−3.4–2.4
HbA1C (%)	27	7.2 (1.1)	6.9 (1.0)	−0.3 (1.0)	−0.7–0.1
CES-D total score	35	7.7 (7.6)	7.0 (7.4)	−0.7 (7.7)	−3.3–1.9
Depressive symptoms (score 16+)		3 (8.6 %)	3 (8.6 %)	Not applicable	Not applicable
No depressive symptoms (score <16)		32 (91.4 %)	32 (91.4 %)		
GAD-7 total score	36	2.7 (2.9)	1.9 (2.6)	−0.7 (2.55)	−1.6–0.1
Anxiety disorder (score 5+)		10 (27.8 %)	7 (19.4 %)	Not applicable	Not applicable
No anxiety disorder (score <5)		26 (72.2 %)	29 (80.6 %)		

Most of the 95 % CIs for the mean score differences included 0, indicating that the changes in the measures from baseline to 6 months were not significant (Table 3). The exception was the PCS score change (SF-12), where we observed a mean increase in score of 3.0 points (95 % CI for the difference, 0.3–5.8), an improvement that is equal to the minimally important difference for interpretation of group mean PCS score differences [80]. The PCS scores for both genders increased, on average, over the 6-month period. The mean decrease in HbA1C levels (for the 27 participants with T1 and T2 measures) is also notable (0.30 %, 95 % CI −0.1–0.7 %), as this is close to the 0.50 % frequently used for judging clinical significance and recognized as somewhat arbitrary [81]. The proportion “at risk” for depressive symptoms (score ≥16 for CES-D) remained the same from baseline to 6 months (9 %), with the majority of participants [33] having the same risk level at both time periods. There was a small decline in the proportion of participants “at risk” of anxiety disorders (score ≥5 for GAD-7), from 28 to 19 % (3 fewer participants “at risk” at 6 months), although half [5] of the participants “at risk” at baseline remained so at 6 months and the 5 that improved (went from “at risk” to “not at risk”) were partially offset by 2 participants who declined (went from “not at risk” to “at risk”). Some trends suggested potential gender differences in self-management behavior. For example, although there was no change in SDSCA scores on average, the SDSCA score increased for males but decreased for females over the 6-month program period. While HbA1C levels declined over the 6 months for both genders, levels remained higher in females compared with males. Also, twice as many females as males were unable to provide HbA1C measures at baseline or 6 months.

We also examined hypoglycemic events and diabetes medication use and found that the majority of participants did not experience changes in these parameters. For 64 % of the sample, there was no change in hypoglycemic events, 8 % experienced a decrease, 11 % experienced an increase, and 17 % were not sure. For 70 % of the sample, there was no change in the use of diabetes medications, 17 % increased use, and 14 % decreased use. At both baseline and 6 months, 36 % of the samples were not taking any diabetes medications.

#### Cost of use of health and social services

The total median costs of the program were $499.00 (Q1–Q3 356.50–602.00) per study participant (see Time 2, “Total diabetes care” costs in Table 4). The direction of the changes in median costs from baseline to 6 months conformed to our hypotheses: median program costs increased (total diabetes care); and median non-program costs either did not change (family physician visits, emergency department visits, ambulance use, home care, other services, supplies and equipment, hospital) or decreased (specialist visits, diagnostic tests, prescription medications). The median cost change in publicly funded (OHIP) health professionals (−6.11) and in other (non-OHIP) health professionals (6.62) offset one another.

**Table 4:** Costs (per patient) of use of healthcare services at baseline and 6 months (*n* = 36, CAD)

Service	Median cost	Q1—Q3 cost	Median cost	Q1–Q3 cost	Difference in median costs
	Baseline	Baseline	6 months	6 months	(6 months—baseline)
Family physician	75.92	75.92–113.88	75.92	37.96–113.88	0.00
Specialist	190.48	64.72–409.27	173.12	22.15–321.25	−17.36
Emergency room visits	0.00	0.00–0.00	0.00	0.00–0.00	0.00
Ambulance service	0.00	0.00–0.00	0.00	0.00–0.00	0.00
Total home care visits	0.00	0.00–0.00	0.00	0.00–0.00	0.00
Total diabetes care^a^	145.00	95.00–214.00	499.00	356.50–602.00	354.00
Other health professionals (OHIP)^b^	6.11	0.00–120.00	0.00	0.00–60.00	−6.11
Other health professionals^c^	71.68	15.00–254.20	78.30	10.00–222.50	6.62
Other services^d^	0.00	0.00–616.00	0.00	0.00–550.00	0.00
Supplies, equipment	0.00	0.00–0.00	0.00	0.00–30.00	0.00
Diagnostic tests	160.62	116.70–279.79	138.50	72.17–269.66	−22.12
Prescription meds^e^	766.60	365.80–1037.43	635.81	392.77–1051.39	−130.79
Acute care hospital	0.00	0.00–0.00	0.00	0.00–0.00	0.00
Total costs	2032.31	1442.73–3563.02	2223.24	1660.16–3546.33	190.93

^a^Includes the costs of kinesiology assessments, group sessions, home/clinic visits, and case conferences

^b^Includes nurse practitioners, nurses, neuropsychologists, pharmacists, mental health counselors, speech and language pathologists, and group programs

^c^Includes dentists, optometrists, podiatrists, chiropodists, foot care nurses, acupuncturists, chiropractors, massage therapists, physiotherapists (not through home care), and audiologists

^d^Includes homemakers, delivered meals, adult day programs, personal trainers, and 911 service

^e^Cost based on amount paid by provincial government and includes dispensing fee

### Secondary objective: primary outcome for RCT

Three outcomes were viewed as potential candidates for the primary outcome for the RCT: SDSCA, HbA1C, and PCS. Going into this feasibility study, we viewed the SDSCA as a strong candidate, given its central role in the theory underpinning the program. However, we found that the SDSCA assessment was difficult to administer for several reasons. The questions use a recommended plan as a reference point for reporting the weekly frequency of consuming fruits and vegetables, monitoring glucose levels, exercising, taking medications, etc., yet some clients had no plan or they were advised to take no medication. This resulted in misinterpretations by clients (e.g., reporting eating no fruits or vegetables during the week even though they did so daily, because they did not have a recommended diet plan) or the lack of applicability of certain questions (e.g., cannot report adherence to medication when none is taken). We also found that the questions relating to glucose monitoring did not apply to a large number of clients, perhaps because they were newly diagnosed with diabetes and glucose monitoring had not yet been recommended to them.

HbA1C was also considered as a potential primary outcome. This measure showed promise in that a reduction approaching clinical significance was observed over the 6 months. While we found that the timing of the measurements relative to the baseline and 6-month benchmarks varied considerably and fell outside a 1–2-week window for some clients, this was due in part to the use of existing client records to obtain these measures (e.g., participants did not provide bloodwork specifically for this study). The overriding concern with this measure was the evidence indicating its poor connection with patient-relevant issues [26].

Finally, we considered the PCS as a primary outcome. This measure appeared more promising, in that participants in our study did not experience difficulty interpreting the SF-12 questions, the summary scores generated from the SF-12 instrument (PCS and MCS) represent well-validated measures of HRQoL, published norms exist for comparison with the Canadian general population, and the PCS score captures physical functional ability which appears to be an important outcome for patients [26]. A recent systematic review found that the PCS was frequently used as a primary outcome in the evaluation of interventions similar to ours for adults with chronic or long-term health conditions [13]. These facts, taken together, suggest that the PCS is the most appropriate outcome for the RCT.

## Discussion

The overarching goal of this feasibility study was to determine the feasibility of conducting a definitive RCT to evaluate an innovative, interprofessional, nurse-led, community-based program to promote increased diabetes self-management in older adults with T2DM and MCC. To address this goal, our primary objective assessed the feasibility of implementing the program in practice. Secondary objectives focused on assessing the feasibility of the study methods, exploring the 6-month change in outcome measures for preliminary evidence of potential effects, and selecting a primary outcome for the RCT.

### Feasibility of the program and study methods

The feasibility of the program was confirmed by the acceptability of the program to providers, peer support volunteers, and participants. There was evidence of “buy in” from all three groups. For example, it was encouraging that participants saw value in the home visits, both to themselves *and* to the healthcare providers. There was recognition that the program provided an improved understanding of the client’s health status and context that was attributed to the combined effect of interprofessional collaboration, synergy among program components, and the unique insights gained from home visits. Further evidence of the acceptability of the program to participants was provided by the high level of engagement for home/clinic visits and group session attendance (97 % for home visits and 76 % for group sessions). Providers and participants requested increased family caregiver involvement, which is supported by research showing that goal alignment (participants, providers, caregivers) leads to increased functional independence and improved symptom management [82]. We showed that client interest in the program was high by exceeding our eligibility, recruitment, and retention targets. We also showed that the study methods were feasible and effective in reaching our target population (e.g., 92 % of the sample had 5+ chronic conditions). We established that the program could be delivered as planned.

We noted that there were some differences between people who completed the program and those who dropped out. Participants who dropped out were more likely to be living with a spouse or someone else, have lower incomes, and be taking more diabetes medications. The latter characteristic may reflect the fact that non-completers had been living with diabetes longer or that their diabetes was more severe or less controlled compared with those who completed the program. This issue will be examined more fully in the RCT.

### Changes in outcome measures at 6 months

The PCS score increased from baseline to 6 months. Our results for HbA1C are within the range of small positive effects (0.24–0.44 % decrease over 6 months) reported in two recent reviews of self-management interventions for people with T2DM [13, 17]. Our results for the PCS are more positive than those included in the reviews, which reported uncertain [17] or no significant effects on HRQoL [13]. Despite the positive change we observed in the PCS, male and female scores were below the Canadian general population norms of 48.1 (9.1) for males and 46.5 (10.2) for females [83]. Lower scores may be due to our focus on a disease cohort but may also relate to age (Canadian norms pertain to the general population and include all ages). MCS scores for the males and females in our study were slightly above the Canadian general population norms of 54.6 (7.7) for males and 53.0 (8.8) for females [83].

We observed no substantial changes in self-management behavior or mental health (MCS, depressive symptoms, or anxiety), whereas the existing evidence shows small positive effects for these variables [13, 17, 84]. These differences may reflect differences in sample size or may be due to the nature of our sample—e.g., most studies excluded older adults and people with MCC, whereas our study included them and consequently may have captured a group of clients more typical of the heterogeneity seen in everyday practice. While our rate for depressive symptoms (9 %) is close to the 11 % reported in the literature [85, 86], our rate for anxiety (28 % at baseline, 19 % at 6 months) was higher perhaps due to the higher morbidity of our sample compared to other study samples.

### Primary outcome for RCT

We evaluated the performance of the SDSCA, PCS, and HbA1C level to determine the most appropriate primary outcome for the RCT. The SF-12 assessment, which provides the PCS, was well received by participants and more reliable and easier to administer, compared with the other measures. Broader support for the PCS exists in a recent systematic review, which found that the PCS was among the most common primary outcomes in interventions similar to ours that were aimed at enhancing self-management of long-term or chronic conditions [13].

### Key lessons learned for the RCT

The feedback from providers, peer support volunteers, and participants was instrumental in identifying proposed changes to the program to ensure its successful implementation in the definitive RCT. The proposed changes were mainly at the operational level and did not change fundamental assumptions underlying the program. There were several important observations made and understandings acquired during the feasibility study that will affect how we implement and evaluate the program in the RCT, including

1. Program administration: The administrative role in the program is considerable, particularly at the beginning, because of the lack of experience with this model of care. There needs to be sufficient support for the administrative role via the provision of an administrative assistant or sharing administrative tasks with other members of the program team.
2. Program synergies: The potential for synergy across program components is high. All three groups indicated this, either by observing the synergies or recommending ways to enhance them. As a result, even though face-to-face contact with clients at home visits was fairly limited (3 h on average over the 6-month program), this was augmented by continuity of the providers over the program period and by multiple points of contact within the program. Both of these features enhanced team collaboration and client relationships and aided in developing a better understanding of the health status and context of all clients. Therefore, efforts should be made to preserve these features of the program in the full RCT, to capitalize on the potential for synergy. These efforts include having all providers present for the entire group session, to ensure consistency in messaging and goal setting. Efforts to shorten the group sessions (all three groups suggested this) should be carefully planned, to maintain the maximum effect of the program. Efforts should also include having all providers trained in key program components, such as the physical exercise element, so that the objectives of these components can be reinforced at all client encounters. Methods of standardizing provider training will be particularly crucial in moving to the larger, multi-site RCT. As in this pilot study, multiple methods will be used to standardize training across sites in the larger RCT, including provider workshops, detailed training manuals, and regular outreach sessions with researchers to identify and resolve challenges in delivering the intervention and ensure that methods of addressing issues are consistent across sites.
3. Peer support volunteers: The need for peer support volunteers may depend on the dynamics of the group sessions. In the feasibility study, peer support volunteers functioned more as participants and less as client supports. Study participants, on the other hand, assumed the role of peer support volunteer as they gained experience with the group sessions. Therefore, the role of peer support volunteer may be unnecessary at sites where participants assume this role or if a close family member or friend attends the group sessions (as suggested by both providers and participants).
4. Case conferences: Case conferences were used primarily to plan the group sessions rather than to discuss client goals and progress. Increased efforts should be made to focus on client goals in all of the program settings, including case conferences, home visits, and the group program.
5. Diabetes within the context of MCC: Although the RN and RD were instructed to focus not only on diabetes care but also on the prevention and management of other chronic conditions, their approach ultimately emphasized diabetes. It is critical to maintain a high level of diabetes care but equally important to move beyond the single-disease focus of the current clinical practice guidelines. The training of diabetes educators should be supplemented with an understanding of the common comorbidities experienced by older adults with T2DM, how these affect diabetes self-management, and self-management strategies that take comorbidity into account. Therefore, the training program for the full RCT and the monthly team meetings should provide more education to providers on resources and self-management strategies appropriate for clients with T2DM within the context of MCC.
6. Medications: The feasibility study considered prescription medications only, yet non-prescription medications can have an important influence on health status, self-management ability, and program effectiveness. Therefore, the full RCT will consider total medications, including over-the-counter medications.
7. Evaluation of program: This feasibility study involved the evaluation of a program implemented in a real-world community-based setting. It included consideration of the acceptability of the program, program fidelity, implementation barriers and facilitators, feasibility of study methods, and a range of potential effects. There were many components to the evaluation, but what seemed to be missing was an overall framework that tied all the pieces together. We reviewed the literature to identify potential conceptual models, with the RE-AIM framework being among the most comprehensive and applicable to our program. The RE-AIM framework guides the measurement of health effects and implementation barriers and facilitators, has been used to measure the pragmatic implementation of healthcare interventions in a variety of real-world settings, and includes the following five dimensions: reach, effectiveness, adoption, implementation, and maintenance [85]. We intend to use the RE-AIM framework to guide the evaluation component of the RCT.
8. Mechanisms underlying the program: The feasibility study did not explore the mechanisms underlying the program, because the small sample size limited the ability to meaningfully explore these mechanisms and because of the need to focus on the more fundamental issue of whether it was feasible to move ahead with a definitive trial. However, our intention is to explore the mechanisms underlying the program in the RCT. Self-efficacy is an important mechanism, according to the social cognitive theory underpinning the program, and the program components were designed to affect different sources of self-efficacy (e.g., group sessions were designed to facilitate social modeling and social persuasion, home/clinic visits used motivational interviewing to encourage and acknowledge mastery of self- management activities). We plan to include a 6-item scale to measure self-efficacy [86] in the RCT, which will allow us to assess whether the program affects self-efficacy, how self-efficacy relates to self-management and HRQoL (PCS), and how self-management relates to HRQoL (PCS). In the RCT, we can also more fully explore the relative contributions of the various program components to determine which ones are responsible for the effects.
9. Subgroup analyses: The small sample size limited our ability to explore differences across subgroups, although the feasibility study provides some initial evidence of characteristics that may influence the effectiveness of the program. For example, there were gender differences in self-management behavior and engagement rates. Providers thought the program would particularly benefit people living alone, and we observed a significant difference in the living status of completers versus non-completers (a higher proportion of completers lived alone). In the RCT, we intend to conduct a comprehensive subgroup analysis to understand who may benefit more from the program. This analysis will consider characteristics including gender, age, social vulnerability, duration with diabetes, and MCC.

### Study strengths and limitations

A particular strength of this study is its focus on a program that is well grounded in theory. Theory is an important consideration in the development of complex interventions, and it enables us to identify several hypotheses that can be tested in the RCT. Face validity of the program was achieved through the involvement of T2DM client and community providers in the design and evaluation of the program. Another strength of this study is that it was undertaken in a real-world setting within the existing healthcare system and using existing resources. As a result, the facilitators and barriers to implementation identified in this study are likely to be indicative of what would occur if the program was implemented in a real practice setting. This study also considered the costs of use of health and social services, which many intervention studies either omit entirely or approach less rigorously.

We should also acknowledge a number of limitations. First, the sample size was intentionally small to allow us to focus on the primary objective, the feasibility of the program. However, this small sample limits the reliability of the statistical analyses and means that the results offer *preliminary/emergent*, not definitive, evidence of the *potential* effectiveness of the program. Second, the study was a single-arm, pre-test/post-test design, which is less rigorous than a RCT. A number of measures were taken in the study to address the common threats to internal validity (placebo effect, history, statistical regression). Confounding with usual care was difficult to address, because usual care was in place in addition to the intervention and these services could account for some or all the changes observed between baseline and 6 months. However, many participants discontinued their regular diabetes clinic visits while receiving the intervention, so it is unlikely that a substantial portion of the effects observed are attributable to usual care. Third, a complete case analysis was conducted, which can result in biased estimates of the results. In the RCT, more sophisticated methods (e.g., multiple imputation) will be used to address missing data. Fourth, while the instruments used in the study have been validated in many populations, this work often excludes complex patient groups such as older adults with MCC. However, the instruments appear to be performing as expected in this study (e.g., PCS scores were lower than the Canadian norms for the general population as expected for a disease cohort involving older adults, rates for depressive symptoms were similar to those reported in the literature). Fifth, we were unable to explore the mechanism through which the program improved HRQoL (PCS). We also did not explore the impact of participant differences, such as distinguishing between people with incident versus long exposure to T2DM or with higher versus lower levels of social vulnerability. These and other differences can result in different risk profiles and management strategies and can impact the effectiveness of the program. In the RCT, we will be conducting a subgroup analysis to explore the differential effectiveness of the program, measuring self-efficacy, and exploring how self-efficacy relates to self-management and the PCS. Sixth, the feasibility study involved a single site. Sites may differ on characteristics that can affect implementation of the program. For example, staffing is known to vary across DECs, with some having an RN and RD (as at the study site) and others having one healthcare professional (such as a pharmacist or RD) [87]. The RCT will involve multiple sites, to explore how the program performs across a broader range of settings.

## Conclusions

This study provides the first evidence of the feasibility of implementing in practice an innovative, interprofessional, nurse-led, community-based program for older adults with T2DM and MCC. Based on the results of this feasibility study, a definitive trial to determine the effectiveness of the program would be feasible in the Canadian population of older adults with T2DM and MCC. Some adaptations to the program and study methods were identified from feedback from the providers, peer support volunteers, and participants. This paper also identified several key lessons learned from the feasibility study that will be carried forward to the RCT.

## Appendix

ᅟ

**Table 5:** Program fidelity checklist items

Type of fidelity	Component	Data sources
Fidelity to theory	Program includes relevant “active ingredients” based on theory	Program protocol, training manual
Provider training	Providers received between 11.5 and 13.5 h of training at the beginning of the program (case conferences, home visits, group sessions, HSEP)	Training protocols and standardized training manuals
	Ongoing supervision provided throughout program period	“Outreach” meeting records
	Periodic training at “outreach” meetings to prevent “drift”	“Outreach” meeting records
Recruiter training	Recruiters receive 1–2 h of training prior to the start of the program, and throughout program as needed	Training protocol, attendance records
Research assistant training	Research assistants received 3 h of training at the beginning of the program and additional training, as needed	Training protocol, attendance records
Program implementation	Standardized training protocol developed	Training protocol
	Strategy exists to avoid contamination (e.g., RN and RD dedicated to program and not providing usual care)	Study protocol
	Delivered “active ingredients” (holistic care, caregiver involvement, inter-professional collaboration, motivational interviewing)	Visit records, case conference reports
	Regular provider monitoring over program period	“Outreach” meeting records
	≥1 case conference for each client	Case conference reports
	≥1 home visit for each client	Visit record
	≥1 group session attended by each client	Group session attendance record
	Provider perception of program	“Outreach” meeting records, 6-month focus group sessions
	Peer support volunteer perception of program	6-month focus group sessions
Treatment receipt	Self-reported client knowledge	6-month interviews
	Client perceptions of program	6-month interviews
Treatment enactment	Client progress during program	Visit record
	Change in self-efficacy	Baseline and 6-month data collection form, effects analysis
	Change in self-management behavior (SDSCA)	Baseline and 6-month data collection form, effects analysis
	Change in HRQoL (PCS, MCS)	Baseline and 6-month data collection form, effects analysis
	Change in HbA1C	Baseline and 6-month data collection form, effects analysis
	Change in CES-D (depressive symptoms)	Baseline and 6-month data collection form, effects analysis
	Change in GAD-7 (anxiety)	Baseline and 6-month data collection form, effects analysis

ᅟ

**Table 6:** Feedback from providers, peer support volunteers and participants

Component	Characteristic	Feedback from feasibility study	Suggested changes for RCT
Program			
Administration	Burden	Program requires considerable time for coordination, communication, and document completion, particularly at beginning [I]^a^	-Emphasize that all team members help RN with administration
			-Provide RN/RD with copies of all forms for all clients at start of program
Case conferences	Length of meeting	Variable over 6-month program (initial meetings took more time, but this decreased as experience with clients/program increased) [I]	-Allow 1 h per month for case conferences, which has proven to be more time than usually required
	Client goals	Minimal discussion of client-centered goals (mainly identified implications for educational content of upcoming group sessions) [I]	-Maintain group focus because preferred by providers and other changes (peer support volunteers at case conferences) will require a more general format (to avoid confidentiality concerns)
	Community service referrals	Minimal discussion of client’s specific service needs (mainly identified information on general services to share in group sessions) [I]	-Modify visit record to capture information on community referrals
Home visits	Challenges of setting	Limited access to clinical information, assessments and resources available at office (e.g., primary chart information, kinesiology assessment forms/handouts, blood pressure monitors, place to record client action items/goals) [I]	-Train providers to perform assessment similar to kinesiologist’s assessment (gait and mobility test called “Timed Up and Go”)
	Provider attendance	Preferable to have both providers (RN, RD), especially for first visit and/or for complex clients (safety concerns, maximize/confirm observations, collaborate on complex care issues) [I]	-Have RN and RD attend the first home visit and up to 50 % of all follow-up visits
	Provider training	Providers requested more training on motivational interviewing, management strategies for common diabetes discordant conditions (e.g., COPD, arthritis) and social determinants of health, information on assessing and recommending activities for frail older adults, and information about the Home Support Exercise Program (HSEP) [I]	-Provide more training on motivational interviewing
			-Revise training manual to include information on theory, common comorbidities, and determinants of health
			-Train program coordinator, RN and RD on HSEP
	Length of time	Variable over the 6-month program (initial meetings longer but decreased with understanding about client’s health status/issues and experience with program) [I]	-Allow 3 h for initial visit and 2.5 h for follow-up visits
	Frequency of visits	Bi-monthly visits worked well for most clients, although a more flexible model that enabled extra visits would benefit some clients [I]	-Allow for a maximum of 3 home visits over 6 months (initial visits, 2 bi-monthly follow-up visits)
	Scheduling	Scheduling was left to the providers, which resulted in delays between baseline interviews and the first home visit, and caused some home visits to be scheduled beyond the 6-month period [I]	-Provide providers with schedule of all home visits and group sessions (for full 6 months) at start of program
Group sessions	Challenges	Group format limits ability to focus on client-centered goals and needs (individual goals/needs too personal for group format) [I]	-Reinforce importance of maintaining group-focus at group sessions in training
		Difficult to ensure that all people, including the quieter individuals, have an opportunity to contribute and that group content is relevant to everyone in group [I, P]	-Ensure that training program and manual reinforces group facilitation skills and importance of maintaining a group focus in group sessions
	Schedule	Schedule that suited clients had following features: mid-day start, education session at end, ≥1 h between meals and physical exercise [I]	-multiple sites will be used in RCT and may have different start times and schedule needs to be structured accordingly
	Length of session	Session should not exceed 3 h and could be less [I, PS, P]	-Shorten group sessions to 2 h
	Attendance by team members	Team members only attended their portion of the group session; it would be better to have the whole team stay for the entire session to ensure consistency [I]	-Recommend that all team members (program coordinator, RN and RD) stay for the entire group session in the RCT
	Physical exercise component	Exercises were not always appropriate for all clients—e.g., exercises need to more varied and to accommodate the wide range of ages and physical abilities [I, P]	-Train RN, RD, and program coordinator in HESP and have them deliver the physical exercise component (HESP consists of basic exercises that everyone can do, and can be adapted to different abilities)
		Better integration of Health Support Exercise Program (HESP) discussed at group sessions with other program components (e.g., providers did not review HESP at home visits because assumed done at group sessions and lacked training) [I, P]	-Train RN and RD on HESP
		Alternatives to the kinesiology assessment should be explored. It is required prior to participation in physical exercise session to minimize the risk of injury, but it delayed start of the group sessions and post-program interviews [I]	-Train providers to conduct similar assessment (a gait and mobility assessment called “Timed Up and Go”)
		Include money in the budget for simple exercise equipment (e.g., Thera-Bands) which were used during the exercise sessions and some clients wanted to continue using these at home [I]	-Use HESP in the RCT, which does not use Thera-Bands (instead uses equipment readily available in the home)
	Program coordinator, physical activity leader	Program coordinator and physical activity leaders requested more information on diabetes [I]	-Recommend that RN/RD be present at entire group session
		Program coordinator indicated that a minimum of 2 h is required for reminder phone calls to clients about upcoming group sessions [I]	-Allow 3 h of time to prepare for and travel to the group session (e.g., make reminder calls, order food etc)
	Peer support volunteers	Motivational interview training is an unrealistic expectation; instead prepare volunteers with questions for use in conversation with clients [I]	-Do not train peer support volunteers on motivational interviewing, just general guidance on support strategies
		Enabling providers to meet volunteers before the program starts could help maximize their synergistic impact [I, PS]	-Provide opportunity for providers to meet peer support volunteers at start of program
		After attending a few group sessions, clients began assuming responsibility for directing the sessions and providing peer support [I, PS]	-This may reduce the need for peer support volunteers at the group sessions for the RCT
		More advanced notice of upcoming group sessions would help facilitate participation of peer support volunteers in the session [PS]	-No changes recommended as this was not a pervasive issue
	Meal component	Educational potential of meal time could be enhanced (e.g., combining snack with educational session to experience different foods, teach balanced snacking, show suitable snacks) [I, P]	-Provide recipes to interested clients (if meals not catered)
			-Provide hardcopy of other diet-related materials as appropriate
		Smaller meals (e.g., soup, sandwiches) preferred by clients and more compatible with exercise component [I, P]	-Serve soup and sandwiches at group sessions (not hot meals)
	Frequency of sessions	Some peer support volunteers thought monthly sessions were too infrequent [PS]	-Retain monthly group sessions in RCT because clients report having many other appointments
	Attendance of family/friends	Potentially beneficial for clients to have family/friends attend (e.g., to encourage client adherence, education for family/friends) [I, P]	-Revise RCT to allow family/friends to attend group sessions
	Transportation	Very few clients required transportation services [I]	-Maintain transportation in RCT as some clients may be coming from rural areas
	Resource materials	Participants indicated a preference for hardcopy handouts rather than referrals to the internet for resource materials [P]	-Recommend to providers to provide hardcopy materials as much as possible

^a^ *I* providers (RN, RD, program coordinator, physical activity leader), *PS* peer support volunteer), *P* participant

ᅟ

**Table 7:** Feedback from research assistants and researchers

Component	Characteristic	Feedback from feasibility study	Suggested changes for RCT
Data collection and analysis			
Baseline and 6-month interviews	Cognitive assessment (baseline)	Montreal Cognitive Assessment (MoCA) was time consuming and more involved than required for confirming informed consent, so an alternative (easier) instrument should be identified	-Replace MoCA with Short Portable Mental Status Questionnaire (SPMSQ) in RCT, which is shorter and easier to administer
	Falls assessment	Providers suggested that a baseline falls assessment would be helpful, with the assessment results available for first home visit	-Suggest providers conduct gait and mobility test (called “Timed Up and Go”) at home visit or group session
	SF-12 assessment	Should be done close to the start of the interview, as per the manual	-No change as SF-12 is located near the beginning of the data collection form
	SDSCA assessment	Assessment difficult to administer: some questions problematic for clients (e.g., red meat consumption, recommended plans) and some questions not applicable to all clients (e.g., glucose monitoring, medications)	-Re-word problematic questions
			-Consider using SF-12 as the primary outcome for the RCT
	HbA1C Levels	HbA1C measures for a number of clients were missing at baseline or 6 months or were taken outside a 1–2-week window relative to the start and end of the program. The importance of recording HbA1C levels at both time periods should be emphasized, as well precision in the timing of taking the measurements	-Emphasize importance of collecting HbA1C levels and ensuring they are timed more precisely relative to baseline and 6 months
	Accuracy of health service use data	When 6-month interviews cross over into a new year, clients need to be reminded to retain the previous year calendars as these were used to provide a more reliable record of health service visits during the 6-month program period	-Issue reminders to clients at any sites in the RCT where the program crosses over into a new year
	Length of time	Baseline interviews longer than 6-month ones (1.5–2 h at baseline, 1–1.5 h at 6 months)	-Allow for different interview times in the RCT (= to those observed in feasibility study)
		Health service data (HSSUI) collection takes longest, especially medication data	-Modify data collection form to include checkbox for diabetes medications
		Could update data collection form with checkbox for common chronic conditions from feasibility study, to expedite collection of chronic conditions data	-Modify data collection form to include a checkbox for common chronic conditions
Home visit documentation	Monthly log sheet	To facilitate progress tracking, home visit log sheets should be faxed weekly to the researchers, rather than submitted monthly	-Maintain weekly/bi-weekly contact between researchers and providers to aid tracking
Home visits, group sessions	Referrals to community services	Referrals to community services are an important element of the program, but it was difficult to track the extent to which referrals were being made, by whom, and follow-up procedures. There needs to be a better way to track referrals	-Include a section in visit record to capture community service referrals
Subgroup analysis	Potential subgroups	Providers suggested the following were “priority clients” for home visits: living alone, recently hospitalized, duration of diabetes ≥15 years, complexity due to multiple chronic conditions or medications	-RCT analysis could explore these characteristics in a subgroup analysis (to see if these clients benefit more than the others)

## Additional file

Additional file 1: STROBE Statement—checklist of items that should be included in reports of observational studies. (PDF 31 kb)

## Abbreviations

CES-D: Center for Epidemiological Studies on Depression (20 items)
DEC: Diabetes Education Centre
DSM IV: Diagnostic and Statistical Manual of Mental Disorders, Fourth Edition
DSME: diabetes self-management education
GAD-7: generalized anxiety disorder (7 Items)
HbA1C: glycemic hemoglobin
HESP (Appendix): Health Support Exercise Program
HRQoL: health-related quality of life
HSSUI: Health and Social Services Utilization Index
MCC: multiple chronic conditions
MCS: Mental Component Summary Score (SF-12)
MoCA: Montreal Cognitive Assessment
PCS: Physical Component Summary Score (SF-12)
RCT: randomized control trial
RD: registered dietitian
RN: registered nurse
SDSCA: Summary of Diabetes Self-Care Activities (11 Items)
SF-12: Short-Form Health Survey (12 Questions)
SF-36: Short-Form Health Survey (36 Questions)
T2DM: type 2 diabetes mellitus
